# Development of Spanish Nutrition Screening Tool for Hispanic Preschoolers

**DOI:** 10.3390/nu16183058

**Published:** 2024-09-11

**Authors:** Denisse Arias, Elaine Lemmon, Marc-Aurel Martial, Mariana Penaranda, Sandra Aguayo, Sarah Gunnell Bellini

**Affiliations:** 1 Nutrition, Dietetics, and Food Science Department, Brigham Young University, ESC S 221, Provo, UT 84602, USA; denisseariasv@gmail.com (D.A.); elaine.h.parry@gmail.com (E.L.); marianapenaranda97@gmail.com (M.P.); sand68aguayo@gmail.com (S.A.); 2 College of Nursing, Brigham Young University, Provo, UT 84602, USA; mamartial@gmail.com

**Keywords:** nutrition screening, malnutrition, obesity, culture, children, preschool, validation, Spanish

## Abstract

Nutrition screening tools may facilitate early nutrition interventions specifically with Hispanic populations that are already at higher risk. There is a paucity of culturally competent and validated nutrition screening tools for 3–5-year-old Hispanic children. The purpose of this study was to develop a Spanish nutrition screening tool for 3–5-year-old children to be used by Spanish-speaking parents in community settings to appropriately address malnutrition risk factors with cultural sensitivity. All phases of the study were conducted in Spanish with native Spanish speakers. Face and content validity were established using focus groups, expert reviews, and pilot testing of the tool with Spanish-speaking parents. Parents of children 3–5 years old whose primary language was Spanish (*n* = 39) completed the nutrition screening, and a nutritionist completed an in-depth nutrition assessment of these children. Criterion validity was measured by comparing the results of the nutrition screening tool with the in-depth nutrition assessment. The nutrition screening tool had a sensitivity of 91.67% and a specificity of 81.48%. The negative predictive value was 69%, and the positive predictive value was 96%. The nutrition screening tool may be used to identify malnutrition in Hispanic children and needs further validation in larger samples.

## 1. Introduction

Latinos comprise 18.9% of the population in the United States (U.S.) and are the largest and fastest-growing minority group [1]. Rates of pediatric obesity have also been growing rapidly among Latino children over the past three decades [2]. From 2015 to 2018, obesity prevalence was 26.6% among Hispanic children compared to 15.5% among non-Hispanic White children [3]. Additionally, the prevalence of obesity according to race or ethnicity estimates Hispanics and non-Hispanic Blacks had higher obesity prevalence compared with their White counterparts at two years of age [4]. Obesity prevalence increases with age, thus efforts towards obesity prevention early in life are crucial to reduce the likelihood of adult obesity and the risk of developing chronic diseases like diabetes and cardiovascular disease [5].

Multiple studies have shown the prevalence and possible risk factors for overweight and obesity in children, providing a broader understanding of the complex nature of obesity treatment [6,7,8,9,10,11,12]. The conditions of the environments where people are born, live, learn, work, play, worship, and grow have an impact on health and can relate to different risks and outcomes in their quality of life [5]. A deeper understanding of the influence and impact of social determinants of health on obesity can facilitate better identification of malnutrition risk. These individual, social, and environmental factors can influence one another and operate throughout childhood and adolescence, leading to weight gain and even escalating to obesity. Some of these factors include food marketing, under-resourced communities, immigration, food insecurity, school environments, access to safe physical activity, and food deserts [5].

Acculturation is an aspect of immigration that may influence obesity risk within the Hispanic population. Acculturation is the process of adopting new practices from the host country through a multigenerational timeline [13]. Obesity among adults of Mexican origin in the U.S. is associated with longer U.S. residence and being born in the U.S. versus Mexico [5], reflecting the negative impact of U.S. dietary patterns.

Another imperative social determinant of health is the language difference affecting Hispanic populations. Spanish is the second most spoken language in the U.S. [14]. Based on data from 2019, 39% of the people who spoke Spanish at home in the U.S. spoke English “less than very well” [14]. This language barrier may contribute to limited health care access and promote higher obesity rates as communication difficulties decrease the quality of care given and motivation to receive care [15].

Identifying children at risk for overweight and obesity early is important to facilitate effective nutrition interventions. A nutritional assessment is a detailed evaluation of a patient’s nutritional status conducted by a dietitian. It is used to diagnose malnutrition and make decisions about recommendations for intervention and treatment of the patient [16]. In contrast, a nutrition screening tool utilizes risk factors to identify at-risk individuals to help make a nutrition diagnosis [17]. Nutrition screening tools are quick and simple tools that any trained healthcare professional or individual can administer.

The Family, Nutrition, and Physical Activity Screening Tool (FNPA) and Nutrition Screening Tool for Every Preschooler (NutriSTEP) were developed in English and translated and validated in Spanish to improve nutrition screening for Hispanic children [18,19]. The FNPA screens for family environment and behavior patterns that lead to increased childhood obesity risk. This tool was validated in a community setting for physicians or school nurses to administer to parents [18]. NutriSTEP assesses the quality of food intake, physical activity, nutrition-related behaviors, and food insecurity. It was also validated in a community setting in Canada for parents to complete [19]. FNPA and NutriSTEP screening tools underwent extensive face and content validation processes to be validated in Spanish; however, they were not originally developed in Spanish. [18,19].

Multiple barriers, such as a lack of culturally appropriate screening tools, language barriers, and lack of access to nutritional assessment and expert consultation, prevent appropriate nutrition screening and nutritional guidance for Hispanic children [20,21,22]. The differing attitudes between first- and second-generation immigrants, levels of acculturation, and shifting cultural patterns in countries of origin warrant a nutrition screening tool for Hispanic children. To prevent the disparity within the Hispanic population and their increased prevalence of obesity, malnutrition, and food insecurity, it is necessary to develop valid, reliable, and culturally competent screening tools that consider the Hispanic population’s cultural background. Validity indicates whether a tool measures what it is supposed to measure and is essential in assessing the performance of the developed tool [23]. The purpose of this study was to develop and validate a culturally sensitive Spanish nutrition screening tool for 3–5-year-old children to be used by Spanish-speaking parents in community settings in the U.S.

## 2. Materials and Methods

### 2.1. Development of Tool

**Figure 1:**
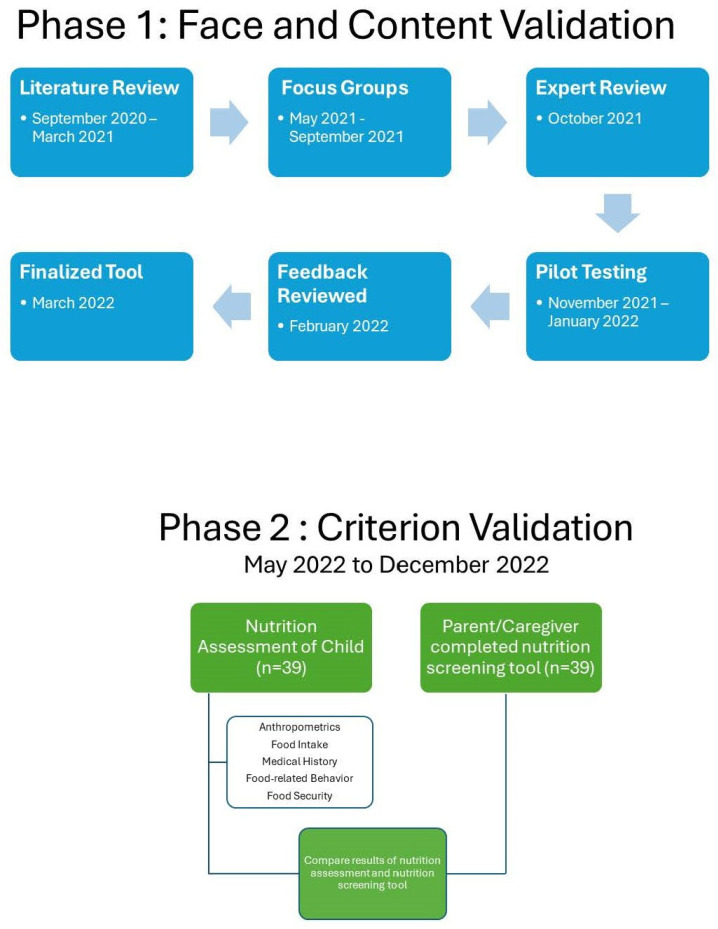
Diagram of phases 1 and 2 of developing nutrition screening tool.

This was a cross-sectional study executed in two phases (Figure 1). Both phases were approved by the Brigham Young University Institutional Review Board. Phase one consisted of face and content validity, and phase two consisted of criterion validation.

### 2.2. Face and Content Validity

Face and content validity were established through a literature review and focus groups with Hispanic parents/caretakers of 3–5-year-olds to create the preliminary screening tool: Cuestionario Rápido de Evaluación Comunitaria en Español (CRECE). The preliminary tool was sent for expert review and pilot-tested with its intended audience. The social-ecological model (SEM) provided the methodological framework for this study [7]. The SEM posits the interaction of intrapersonal, interpersonal, and community factors that influence individual and population health and behavior outcomes [24,25]. Health behaviors of Hispanic children are influenced by individual taste preferences; foods served at home, school, or daycare; cultural norms; acceptable food types; and quality of food available based on socioeconomic status.

The focus groups’ primary purpose was to highlight the common Spanish words and phrases used and discover Hispanic parents’/caretakers’ attitudes and feelings toward various health behaviors. Focus group participants (*n* = 11) were recruited by word of mouth or flyers posted in their community. Focus group meetings were held virtually in Spanish and ranged from 45 to 90 min. To begin, the facilitator, a native Spanish speaker, and a scribe introduced themselves, obtained verbal consent, and presented the study’s purpose. Audiovisual data were recorded using Zoom software (Zoom, 2022 Version 5.9.1), and field notes were documented during and after each meeting. Sonix software (Sonix, 2022) was used for transcription. The focus group questions were divided into four sections: general perceptions of nutrition and health, food security, dietary intake, and behavior. Focus group participants were compensated with an electronic USD 25 Amazon gift card.

A Spanish-speaking researcher verified the focus group transcriptions with the audio recording. Two Spanish-speaking researchers independently reviewed transcripts, and a third Spanish-speaking researcher verified the findings. The research team met to discuss focus group participant responses and created CRECE questions following the multi-step verification process [7].

Next, CRECE was sent to Spanish-speaking pediatric healthcare workers: dietitians (*n* = 3), a nurse (*n* = 1), and medical assistants *(n* = 3). These expert reviewers measured the relevancy, clarity, and simplicity of the tool. A question with a combined Content Validity Index (CVI) score greater than or equal to 0.8 was accepted, between 0.7 and 0.79 was revised, and less than 0.69 was eliminated [26].

**Figure 2:**
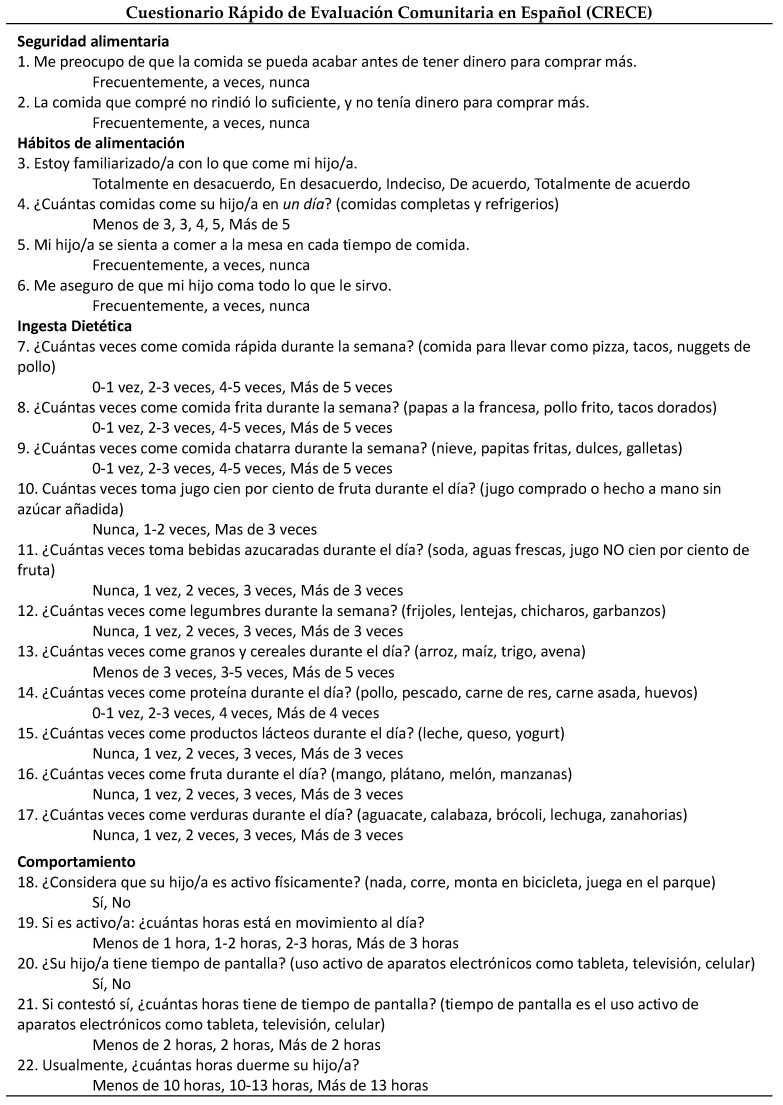

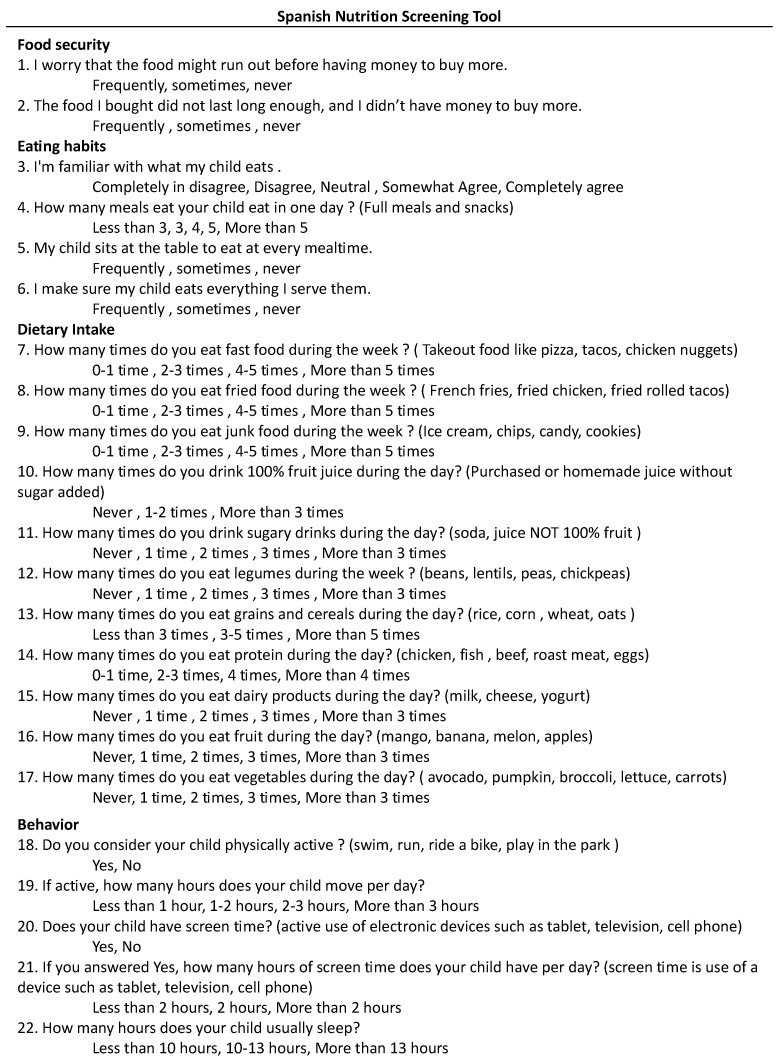
Spanish malnutrition screening tool for children 3 to 5 years old.

Hispanic parents of children 3 to 5 years old *(n* = 23) pilot tested CRECE for clarity and feasibility. Feedback from the pilot test was incorporated into CRECE, such as minor word revisions and a wording change of the food security questions. The first tool consisted of 21 questions. The finalized CRECE was a simple and quick (<10 min) tool in Spanish that a parent could complete and consisted of 22 items and 4 sections: food security (2), food habits (4), food intake (11), and food behavior (5) (Figure 2).

### 2.3. Criterion Validation

The criterion validation phase of the study was carried out between May and December 2022. This study used a complete nutrition assessment, the preferred tool for criterion validity, as the reference standard against the results of CRECE [27]. A convenience sample of Spanish-speaking parents was recruited by word of mouth and emails sent to local Early Learning Essentials participants. Parents/caregivers were included if the primary language spoken at home was Spanish, and they had a child between 3 and 5 years old; no other nutritional, weight, exercise, education, or income criteria were considered for exclusion. The parent/caregiver completed CRECE prior to the full nutrition assessment of the child. Participants provided consent and parental permission for the study.

The full nutrition assessment of the child was completed on site at a community center or campus nutrition assessment lab by trained professionals. The nutritionist assessed adequacy of food intake, anthropometrics, medical history, behavior, and food security status. Food intake was obtained through a 24-h recall by a native Spanish speaking nutritionist and analyzed in Food Processor SQL v11.11 for dietary levels of sodium, added sugar, saturated fat, and the quantity of the U.S. Department of Agriculture (USDA) food groups (ESHA Research, 2017) [28]. Medical history intake included current medical conditions, previous surgeries, hospitalizations, or severe accidents or lesions. Behavior and physical activity status included questions about screen time, sleep patterns, food preferences, and mealtime battles and the time and intensity of physical activity. Food security was measured using a validated two-question food insecurity screening tool [29]. Weight was measured using a SECA 213 (Hamburg, Germany) electronic scale to the nearest 0.1 kg, height was measured using a SECA 882 (Hamburg, Germany) portable stadiometer to the nearest 0.1 cm, and mid upper-arm circumference (MUAC) was measured using a flexible tape. All measurements followed the NHANES procedures [30]. BMI and MUAC z-scores were calculated using Pedi Tools [31]. The cutoffs for BMI and MUAC were based on the Academy of Nutrition and Dietetics/American Society of Parenteral and Enteral Malnutrition Consensus Guidelines and the Center for Disease Control definitions for overweight and obesity [32,33].

**Figure 3:**
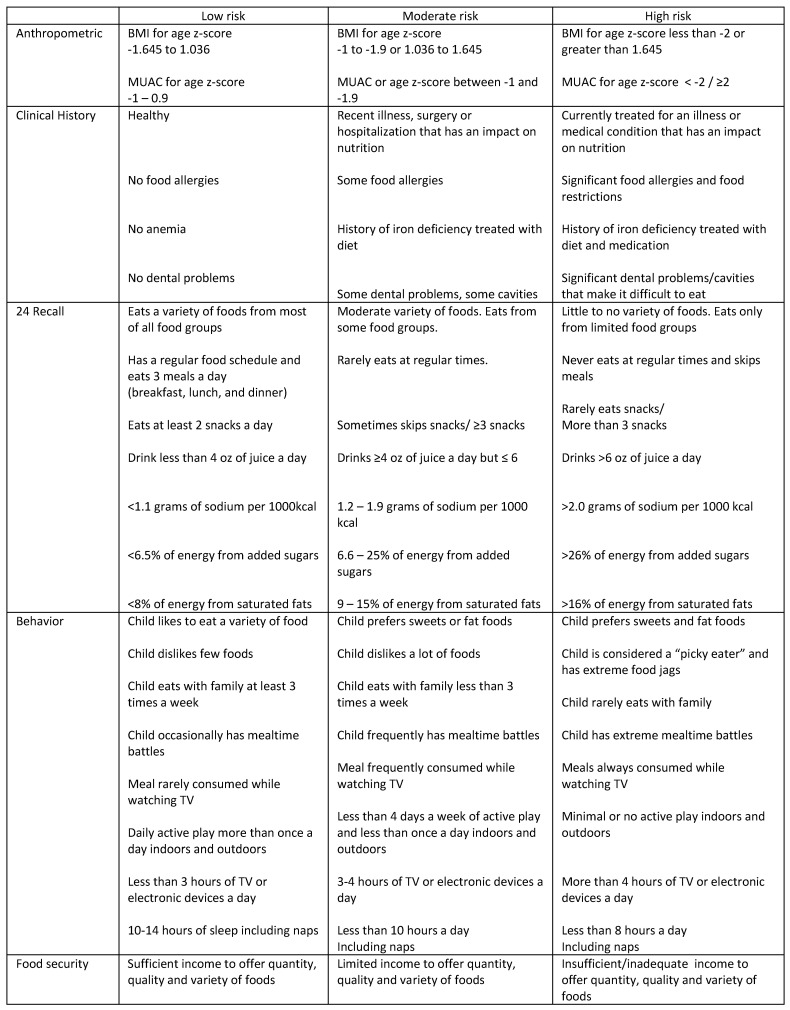
Nutrition Assessment Risk Rating Classification.

For consistency, all assessments were reviewed by a single nutritionist with native Spanish-speaking background. The nutritionist followed an established nutrition assessment risk classification guide (Figure 3). The risk-rating classification guide was developed based on the literature review that included the NutriSTEP risk-rating classification and clinical experience [19]. Based on the risk-rating classification, the child was assigned to low, moderate, or high risk for malnutrition. For anthropometrics, z scores for BMI and MUAC were assessed and weighted individually, while all other components were weighted as one, for a total of 6 points (anthropometrics (2), medical history (1), 24-h recall (1), behavior (1), and food security (1)). If a child received a score of 3 or more at high risk, the child was classified as high risk; if a child received a score of 3 or more at moderate risk, the child was classified as moderate risk; and if either criterion was not met, they were classified as low risk. For example, if a child had one anthropometric component at moderate risk, the other anthropometric component at low risk, medical history at low risk, 24-h recall at moderate risk, behavior at low risk, and food security at moderate risk, then the child was classified as moderate risk for malnutrition. The nutritionist then determined the overall nutrition risk of the child using the nutritionist assessment risk-rating classification. The following parameters were used to categorize CRECE scores: low risk for malnutrition 0–15; moderate risk 16–29; and high risk 30–48.

### 2.4. Data Analysis

Descriptive analysis was used for the demographic data. CRECE results were compared to the nutrition assessment risk rating classification using chi-square to determine sensitivity, specificity, and positive and negative predictive value of the screening tool. Cut points for interpreting sensitivity, specificity, and positive and negative predictive value were excellent at 90% to 100%, good at 80% to 90%, fair at 70 to 80%, and insufficient at 60% to 70%. A *p* value < 0.001 was considered significant.

## 3. Results

The characteristics of the parents/caregivers in phases 1 and 2 are listed in Table 1. Of the 39 parent–child dyads in the criterion validity phase, 53.8% of the parents were between 31 and 35 years of age, and most of the participating parents were females (89.7%). Forty-six percent of participating parents were from Mexico, 17.9% from Colombia, 15.3% from Chile, 10.2% from Peru, and 12.5% from other Latin American countries. Fifty-six percent of parents had lived 5 years or more in the U.S. The educational level of most of the parents (76.9%) was a bachelor’s degree, and the income of the people living in the household for most families was between USD 20,000 and USD 39,000 (33.3%). The mean age of children for whom a full nutrition assessment was completed was 50.54 ± 9.53 months. Nineteen boys and twenty girls were assessed, with a mean BMI z score of −0.15 ± 1.59.

Table 2 shows the comparison between the results of the CRECE nutrition screening tool completed by parents/caregivers and the results of the nutrition assessment completed by the nutritionist. The sensitivity of the tool was 91.67% (SD ± 8.09), and the specificity of the tool was 81.48% (SD ± 7.5%), *p* < 0.0001. The positive predictive value of the CRECE was 69%, and the negative predictive value of CRECE was 96%.

## 4. Discussion

CRECE was created to meet the language and cultural needs of the Hispanic population to accurately capture risks of malnutrition in Hispanic children. The CRECE nutrition screening tool had excellent sensitivity (91.67%) and good specificity (81.48%) [34]. Thus, the tool is good at correctly identifying patients with and without malnutrition. The positive predictive value of the nutrition screening tool indicates that 69% of the children who were classified as being at moderate risk of malnutrition were truly malnourished. The negative predictive value of CRECE indicates that 96% of the children who were classified as being at low risk of malnutrition were truly healthy children with a lower risk of malnutrition. The sensitivity and specificity of the CRECE nutrition screening tool in this study is higher than NutriSTEP, which had a sensitivity of 84% and specificity of 46% for moderate risk [19]. However, caution must be used when interpreting the sensitivity and specificity due to the small sample size.

The reference standard, a comprehensive nutritional assessment, is limited, with some subjectivity and bias of the nutritionist performing the assessment. Therefore, steps were taken to standardize the nutrition assessment and decrease subjective measurements. Components of the nutritional assessment for this study aligned with the American Academy of Pediatrics (AAP) recommendations for evaluating and treating overweight and obesity in children [5]. This included BMI, medical history, physical activity, family and home environment factors, intake of sugar-sweetened beverages, portion sizes, snacking behavior, screen time, sedentary behavior, sleep duration, and food security [5].

One of the main strengths of this nutrition screening tool is its development in Spanish and its consideration of culturally competent aspects such as cultural background, values, attitudes, and beliefs. Research team members included a nutritionist from Mexico, a Hispanic medical assistant, and three dietetic students from Mexico and Bolivia. Researchers conducted focus groups in Spanish with participants from Mexico, Honduras, and Venezuela. The feedback from persons from different Spanish-speaking countries aided in creating well-understood questions. Spanish-speaking researchers verified and reviewed the focus group transcriptions with the audio recording. An interdisciplinary and Spanish-speaking expert panel reviewed the nutrition screening tool. The data included in the nutrition assessment were obtained by trained native Spanish speakers, and the nutrition assessment was conducted by a nutritionist from Mexico. Participants in the criterion validation phase were from different Hispanic cultures, with 46% of the participants being from Mexico, 18% from Colombia, 15% Chile, and 10% from Peru, which gave a broader understanding of the attitudes and beliefs of Hispanic culture.

A culturally competent validated CRECE may facilitate individuals receiving the right nutrition intervention. This becomes more important with populations that are already at higher risk of being overweight, obese, and developing related comorbidities, such as in the Hispanic population [5]. Additionally, minority populations have less access to higher-level interventions such as a nutritional assessment and therefore benefit from an affordable, easy-to-use, and replicable screening tool that can create awareness and appropriately direct to next steps [21].

The nutritional assessment for this study was detailed and comprehensive, while CRECE was quick and easy to use. Although CRECE was completed by parents, it had a similar predicting capacity to an expert’s nutritional assessment. Additionally, it did not require anthropometrics and took 10 min to complete, as opposed to an approximately 25 min nutritional assessment, making it easy to complete in a community setting.

CRECE was created electronically for easy accessibility and rapid convenience in the home environment. Overall, 96% of the Hispanic households reported usage of computers, and 93% of Hispanic households had smartphones [35]. The electronic design of CRECE permits easy access at home by parents or other family members. Simultaneously, it can be used prior to healthcare professional visits or for telehealth visits. Telehealth visits have increased in the overall adult population in the U.S. [36]. CRECE could break barriers and facilitate opportunities for the Hispanic population.

A major limitation of this study is that the sample size is smaller compared to other validation studies [18,19,37]; therefore, the tool should be used with caution and requires further testing. Development of the screening questions may have been biased due to focus group participants’ desire to please an authority figure. Another limitation is the nutritional assessment and nutrition screening tool only identified Hispanic children at low and moderate risk. Most participants in this study were from Mexico, which mirrors U.S. demographics; however, most of the participants had a bachelor’s degree, and 18% had an annual income of more than USD 100,000, unlike U.S. demographics [1]. The CRECE nutrition screening tool may not be valid in other Hispanic populations.

## 5. Conclusions

The CRECE nutrition screening tool can act as a preventative measure and allow for early nutrition intervention. Screening for nutrition risk in Hispanic children creates awareness of the social determinants of health that could play a role in malnutrition and can appropriately direct the child to next steps and a referral to a professional. The CRECE nutrition screening tool in this study takes into consideration culture and language and was created specifically for Hispanic children in the U.S. The CRECE nutrition screening tool proved to be high in sensitivity and specificity and truly detected Hispanic children at risk and without risk of malnutrition. Further validation studies with larger samples are needed to evaluate the effectiveness of this tool in various populations and settings.

## Figures and Tables

**Table 1:** Demographics of parents/caregivers who participated in focus groups (phase 1) and who completed nutrition screening in phase 2 (criterion validation).

	Phase 1 N = 11	Phase 2 N (%)
Spanish as primary language		39 (100)
Identified as Latin		39 (100)
Age		
20–25	-	1 (2.5)
26–30	3	11 (28.2)
31–35	5	21 (53.8)
36–40	2	5 (12.8%)
41–45	1	1 (2.5)
Gender		
Males	4	4 (10.2)
Females	7	35 (89.7)
Country of origin		
Mexico	8	18 (46.1)
Colombia	-	7 (17.9)
Chile	-	6 (15.3)
Peru	-	4 (10.2)
Puerto Rico	-	1 (2.5)
Dominican Republic	-	1 (2.5)
Uruguay	-	1 (2.5)
Honduras	2	1 (2.5)
USA Venezuela	- 1	1 (2.5) -
Time living in the USA		
>5 years	-	22 (56.4)
<1 year	-	7 (17.9)
1–2 years	-	6 (15.3)
3–4 years	-	4 (10.2)
Most spoken language at home		
Spanish	1	35 (89.7)
Mix of English and Spanish	10	4 (10.2)
Marital Status		
Married	11	36 (92.3)
Single	-	2 (5.1)
Divorced/Separated	-	1 (2.5)
Educational level		
Bachelor’s degree	5	30 (76.9)
Some college	1	4 (10.2)
High school diploma	1	3 (7.6)
Advanced degree	3	2 (5.1)
Annual income of the people living in the household (USD)		
Less than 19,000	1	7 (17.9)
20,000–39,000	4	13 (33.3)
40,000–59,000	3	10 (25.6)
60,000–79,000	2	2 (5.1)
More than 100,000	1	7 (17.9)

**Table 2:** Comparison of Spanish nutrition screening tool against nutrition assessment.

Spanish Nutrition Screening Tool	Nutrition Assessment		
	Low Risk	Moderate Risk	Total *(n*)
Low risk	22 TN	1 FN	23
Moderate risk	5 FP	11 TP	16
Total *(n*)	27	12	39

TN = true negative; FN = false negative; FP = false positive; TP = true positive.

## Data Availability

Researchers do not have permission to share data.
